# Self-standing heterostructured NiC*_x_*-NiFe-NC/biochar as a highly efficient cathode for lithium–oxygen batteries

**DOI:** 10.3762/bjnano.11.163

**Published:** 2020-12-02

**Authors:** Shengyu Jing, Xu Gong, Shan Ji, Linhui Jia, Bruno G Pollet, Sheng Yan, Huagen Liang

**Affiliations:** 1 School of Information and Control Engineering, China University of Mining and Technology, Xuzhou, Jiangsu 221008, China; 2 College of Biological, Chemical Science and Chemical Engineering, Jiaxing University, Jiaxing, 314001, China; 3 Low Carbon Energy Institute, China University of Mining and Technology, Xuzhou, Jiangsu 221008, China; 4 Hydrogen Energy and Sonochemistry Research Group, Department of Energy and Process Engineering, Faculty of Engineering, Norwegian University of Science and Technology, NO-7491 Trondheim, Norway; 5 Shanghai Time Shipping CO., LTD, Shanghai, 200126, China

**Keywords:** electrocatalytic performance, lithium–oxygen batteries, N-doped carbon, nickel carbide, oxygen evolution reaction (OER), oxygen reduction reaction (ORR), specific capacity

## Abstract

Lithium–oxygen batteries have attracted research attention due to their low cost and high theoretical capacity. Developing inexpensive and highly efficient cathode materials without using noble metal-based catalysts is highly desirable for practical applications in lithium–oxygen batteries. Herein, a heterostructure of NiFe and NiC*_x_* inside of N-doped carbon (NiC*_x_*-NiFe-NC) derived from bimetallic Prussian blue supported on biochar was developed as a novel self-standing cathode for lithium–oxygen batteries. The specific discharge capacity of the best sample was 27.14 mAh·cm^−2^ at a stable discharge voltage of 2.75 V. The hybridization between the d-orbital of Ni and s and p-orbitals of carbon in NiC*_x_*, formed at 900 °C, enhanced the electrocatalytic performance due to the synergistic effect between these components. The structure of NiC*_x_*-NiFe-NC efficiently improved the electron and ion transfer between the cathode and the electrolyte during the electrochemical processes, resulting in superior electrocatalytic properties in lithium–oxygen batteries. This study indicates that nickel carbide supported on N-doped carbon is a promising cathode material for lithium–oxygen batteries.

## Introduction

Clean and sustainable renewable energy sources, such as wind and solar, account for a slowly growing fraction of the energy that is consumed worldwide [1–2]. Due to the unstable and intermittent power output of most renewable energy sources, energy storage and conversion devices play an important role in providing electricity in an efficient, constant, on-demand, and reliable manner [3–6]. Oxygen reduction reaction (ORR) and oxygen evolution reaction (OER) play critical roles in many clean energy storage and conversion devices (e.g., hydrogen produced from water splitting via water electrolyzers, hydrogen fuel cells, and metal–air batteries [7–10]). In order to meet the requirements for efficient catalysts in practical applications, platinum group metal (PGM)-based catalysts are currently used as principal catalysts to reduce the overpotential of ORR and OER due to their slow kinetics [11–13]. The high cost, poor poisoning tolerance, and scarcity of PGM-based ORR and OER catalysts significantly impede their application in energy storage and conversion devices at a large scale [14–15]. Therefore, there is an urgent and high demand for the development of alternatives to these PGM-based catalysts, at low cost and with fairly high ORR and OER activities.

Significant progress has been made in the development of alternative ORR and OER catalysts, such as transition metal oxides [16–18], heteroatom-doped carbons [19–20], and transition metal nitrides and carbides [21–23]. Due to their surface physicochemical properties (similar to PGMs), high durability, and high electrical conductivity, transition metal carbides have recently become an active research topic. As such, they have been intensively studied as promising alternatives to PGM catalysts in the development of alternative ORR and OER catalysts [24–25]. The hybridization between the d-orbital of the transition metal and s- and p-orbitals of carbon effectively stretch the d-band structure of the transition metal. This results in a similar d-band of PGMs, which makes these metal carbides promising candidates to replace PGM-based ORR and OER catalysts [26]. For instance, molybdenum/tungsten carbide with a well-defined nanostructure was synthesized via a hydrothermal method, and the obtained metal carbide catalyst yielded a high capacity in lithium–oxygen batteries [15]. Also, titanium carbide was synthesized by Bruce et al. as cathode material for lithium–oxygen batteries. It significantly reduced the overpotential and exhibited high efficiency towards ORR and OER [27]. Mu et al. reported that 2D niobium carbide (NbC) nanosheets exhibited enhanced catalytic activity and durability towards ORR [28]. Although progress has been made in the development of transition metal carbides towards ORR and OER, their catalytic performance still needs to be greatly improved to meet the requirements for practical applications. The synergistic effect between transition metal carbides and carbon materials can further enhance the catalytic properties regarding ORR and OER [29–32]. Many metal carbides encapsulated in N-doped graphitic carbons have been developed and have attracted much research interest due to the Pt-like electronic structure and high catalytic activity towards ORR and OER [33]. FeC, Co_3_C, WC, and Mo_2_C (MC) wrapped or supported by N-doped carbons (N-C) have been successfully developed as catalysts for Li–O_2_ batteries. These catalysts show enhanced electrocatalytic activity with good stability [33–35]. Among these MC@N-C materials, FeC@N-C catalysts exhibited the best catalytic activity towards ORR and OER. Therefore, to develop low-cost FeC@N-C catalysts with high catalytic performance is desirable to replace the conventional PGM-based ORR and OER catalysts.

Bimetallic Prussian blue analogues (PBAs) are metal organic framework (MOF) materials, which are promising precursors to prepare transition metal carbides with a porous cubic nanostructure exhibiting excellent catalytic properties [36–37]. Biomass materials can be used to prepare N-doped carbons since their proteins contain nitrogen atoms. This method is an economically viable way to produce N-doped carbons at a large scale [38–39]. Our group has synthesized a series of 3D self-standing electrodes [40–43] by depositing MOFs on biomass followed by either a carbonization or a phosphating step. These electrodes can be directly used as cathodes in Li–O_2_ batteries. In this work, the NiFe-PBA/pomelo peel (PP) precursors were prepared in a similar way as in the previous literatures [40–42]. However, a hydrothermal process was introduced here as a new treatment step prior to the precursor calcination in order to modify the properties of the prepared electrode materials. Heterostructured NiC*_x_*-NiFe-NC derived from bimetallic Prussian blue supported on biochar was synthesized for the use in Li–O_2_ batteries. The electrocatalytic properties of the obtained electrodes were evaluated in a Li–O_2_ battery and these electrodes showed superior catalytic performance in Li–O_2_ batteries.

## Experimental

### Preparation of NiFe-PBA/PP-T

NiFe-PBA/PP precursors were prepared based on [40–42]. Several pieces of NiFe-PBA/PP precursors were immersed in a NaOH solution containing anhydrous ethanol (20 mL) and 0.025 mol·L^−1^ NaOH aqueous solution (30 mL). The obtained solution was transferred to a Teflon-lined autoclave and heated to 100 °C for 30 min. The obtained products were washed with ethanol and water alternatively and calcined at a certain temperature for 2 h with a heating ramp of 5 °C·min^−1^ in a tube furnace in nitrogen atmosphere. The calcined samples were labeled as NiFe-PBA/PP-T.

### Physical characterization

X-ray diffraction (XRD) patterns were recorded using a PANalytical B.V. Empyean X-ray diffractometer with Cu Kα radiation (λ = 1.5406 Å). The surface morphology of the film catalyst was studied via scanning electron microscopy (SEM) on a Carl Zeiss Ultra Plus scanning electron microscope. Transmission electron microscopy (TEM), high-resolution TEM (HR-TEM), and energy-dispersive X-ray spectroscopy (EDX) were carried out on a FEI Tecnia G2 F20 high-resolution transmission electron microscope operating at 200 kV. The surface composition of the samples was analyzed by X-ray photoelectron spectroscopy (XPS) on a ESCALAB 250Xi electron energy spectrometer using Al Kα radiation.

### Electrochemical measurement

The electrochemical performance of the obtained products was evaluated in CR2025 coin cells with 17 holes, each with a diameter of 1.0 mm, on the cathode side. The obtained products were directly used as the binder-free air cathode, lithium foil was used as the anode, and LiCF_3_SO_3_ dissolved in tetraethylene glycol dimethyl ether was used as the electrolyte. A glass filter (Whatman grade GF/D) was used as the separator in these coin cells. The cell assembly was carried out in an Ar-filled glove box (H_2_O < 0.5 ppm, O_2_ < 0.5 ppm). The sealed coin cells were placed in a glass bottle filled with pure oxygen. The discharge/charge was carried out in a cell voltage range from 2.0 to 4.5 V (vs Li^+^/Li) at room temperature on a battery tester (Neware, CT-3008, China). The specific capacity was calculated using the mass of the entire cathode. Electrochemical impedance spectroscopy (EIS) in a frequency range from 100 kHz to 0.1 Hz and with a perturbation amplitude of 5 mV was used on the PARSTAT 4000 (AMETEK, USA) electrochemical workstation. Cyclic voltammetry (CV) was performed at a scan rate of 0.1 mV·s^−1^ and in a cell voltage range from 2.0 to 4.5 V vs Li^+^/Li.

## Results and Discussion

**Figure 1:**
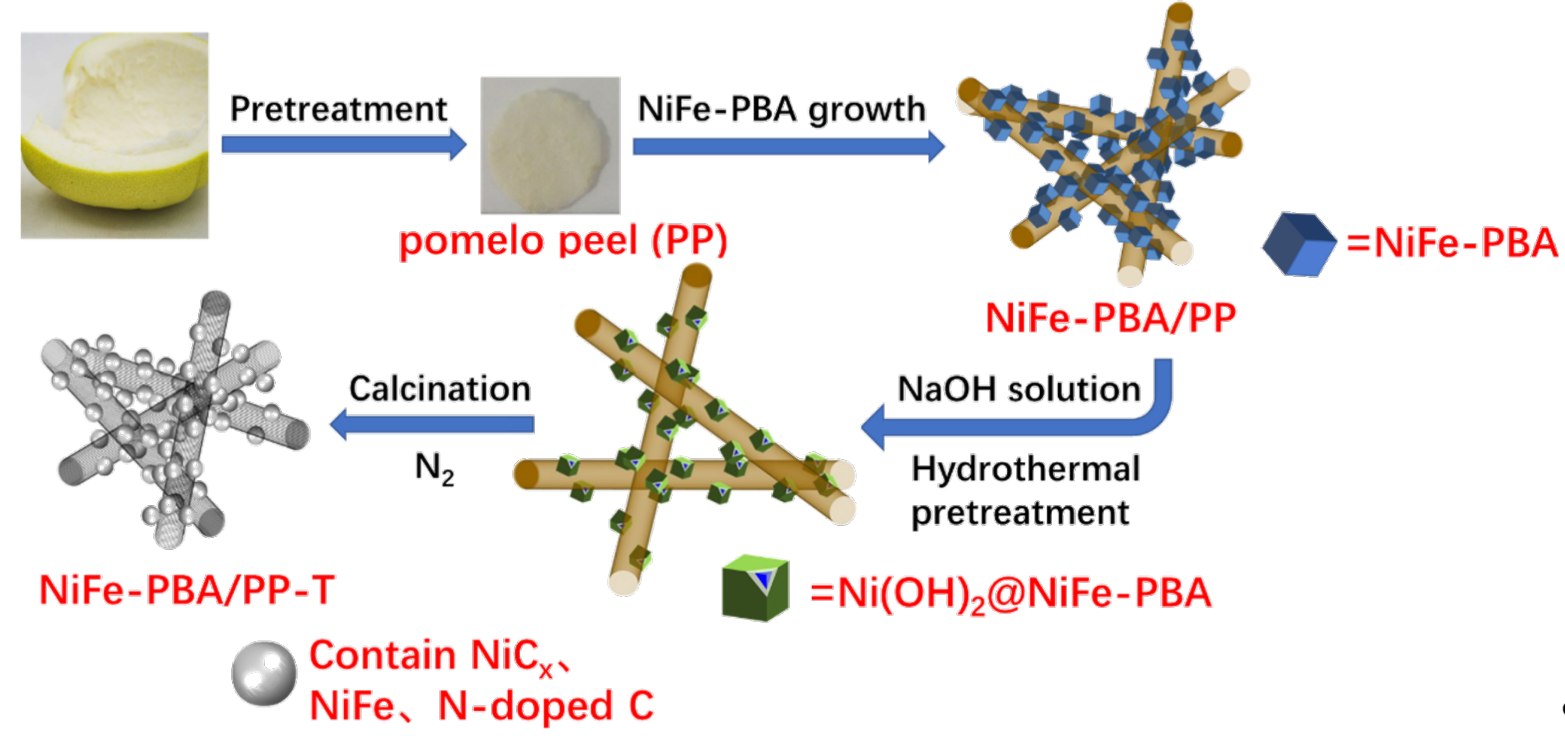
The strategy used for synthesizing 3D free-standing NiFe-PBA/PP-T cathodes. (Adapted from [42], Copyright © 2018, with permission from Elsevier).

**Figure 2:**
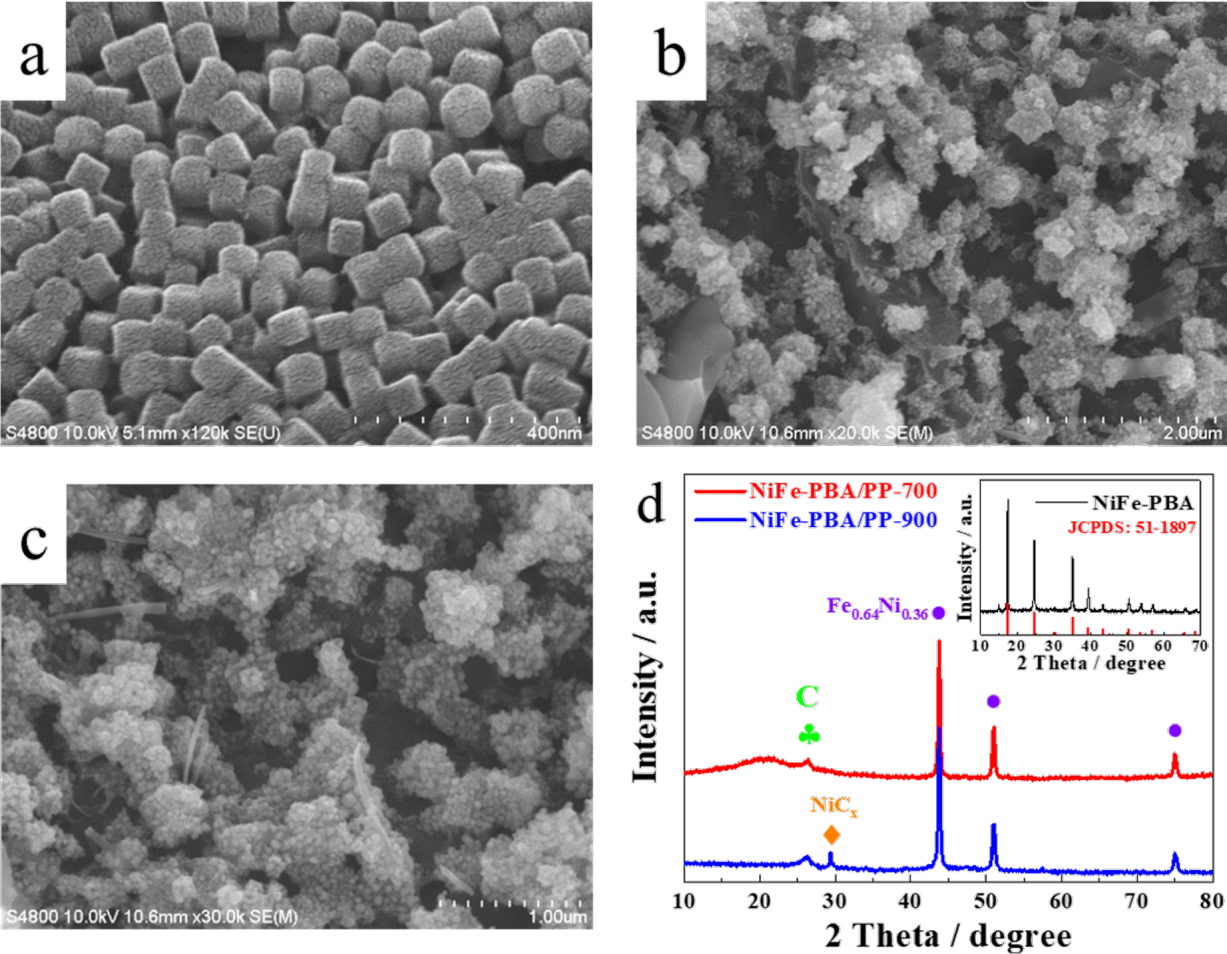
(a) SEM images of NiFe-PBA/PP, (b) NiFe-PBA/PP-700, and (c) NiFe-PBA/PP-900. (d) Corresponding XRD patterns of NiFe-PBA/PP-700 (red curve), NiFe-PBA/PP-900 (blue curve), and of NiFe-PBA (inset, black curve).

The overall process of synthesizing NiFe-PBA/PP-T is schematically illustrated in Figure 1. Initially, the freshly cut pomelo peel was mixed with Na_3_C_6_H_5_O_7_, Ni(NO_3_)_2_, and K_3_[Fe(CN)_6_] to form bimetallic Prussian blue analogues with a MOF structure at room temperature. Ni_3_[Fe(CN)_6_]_2_ precipitate was formed when Ni(NO_3_)_2_ and K_3_[Fe(CN)_6_] were mixed. During the formation of Ni_3_[Fe(CN)_6_]_2_, Ni ions and N atoms from the hexacyanoferrate [Fe(CN)_6_]^3−^ cross-link, which results in the formation of a three-dimensional and cubic framework with abundant Ni, Fe, N, and C within the structure. As shown in the inset of Figure 2d, the main diffraction peaks of the Ni–Fe PBA precursor are consistent with the standard patterns of Ni_3_[Fe(CN)_6_]_2_ (JCPDS: 51-1897). During a hydrothermal pretreatment, the ion-exchange reaction of OH^−^/[Fe(CN)_6_]^3−^ occurred at the interface between NiFe-PBA cubes and the NaOH solution, resulting in a Ni(OH)_2_/NiFe-PBA core–shell structure [44–46]. During the calcination process, Ni(OH)_2_ was converted into NiC*_x_*, and the NiFe-PBA core was converted into a NiFe alloy coated with N-doped carbon.

The microstructure of NiFe-PBA/PP-T was evaluated by SEM. Figure 2a shows that the Prussian blue analogues formed on the surface of PP have a well-defined cubic shape with an uniform particle size distribution. The particle size was approx. 80 nm. These cubic particles were made out of many small particles and many cavities were observed on the surface. SEM images of NiFe-PBA/PP-700 and NiFe-PBA/PP-900 (Figure 2b and Figure 2c) show that, after the sample was calcined at a high temperature, the cubic-shaped particles disappeared and were replaced with aggregated irregular particles formed on the surface of the carbon support material. Compared to the sample calcined at 700 °C, NiFe-PBA/PP-900 had a grape-like morphology (Figure 2c). The crystal structure of NiFe-PBA/PP-T samples was obtained from XRD (Figure 2d). A characteristic peak at approx. 2θ = 26.4° was observed in the XRD patterns of both samples, and was attributed to the (002) plane of graphite (JCPDS No: 41-1487). In the XRD pattern of NiFe-PBA/PP-700, three peaks at 43.6°, 50.8°, and 74.7° were attributed to the (111), (200), and (220) planes of the Awaruite phase of the Fe_0.64_Ni_0.36_ alloy (JCPDS No: 47-1405). After the sample was heated to 900 °C, the three peaks of the Fe_0.64_Ni_0.36_ alloy remained, and a new peak at 29.4° was observed and indexed to the (111) plane of NiC*_x_* (JCPDS: 45-00979). In other words, nickel carbide was formed during the calcination process, which indicates that NiC*_x_* was formed at 900 °C.

**Figure 3:**
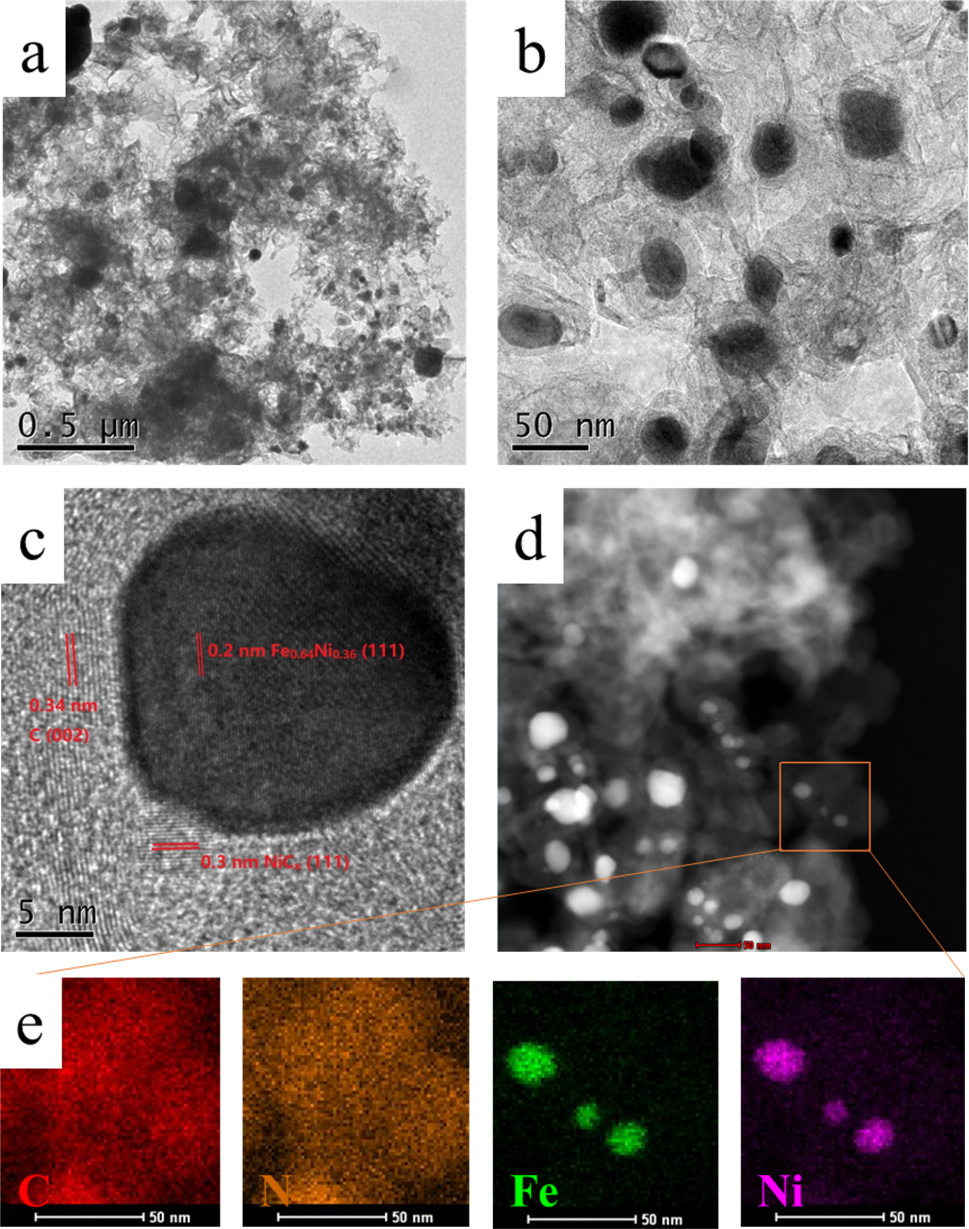
(a–c) HR-TEM images and (d,e) element mappings of NiFe-PBA/PP-900.

The detailed structure of NiFe-PBA/PP-900 was further investigated by HR-TEM. A flocculent morphology of NiFe-PBA/PP-900 was observed in the HR-TEM image (Figure 3a), and was found to be composed of many irregular sheet-like materials. In the zoomed HR-TEM image (Figure 3b), many cavities and wrinkles are clearly observed in the carbon material. Moreover, irregular black particles were formed at the surface, which had intimate contact with the carbon material. Well-defined lattice fringes were also observed in the HR-TEM image (Figure 3c). The measured *d*-spacing of the lattice in the black particle was approx. 0.20 nm, and it was assigned to the (111) plane of Fe_0.64_Ni_0.36_. The black particle shown in Figure 3c was tightly surrounded by lattice fringes, and the measured *d*-spacing of these lattices was approx. 0.30 and 0.34 nm, which corresponded to the (111) plane of NiC*_x_* and to the (002) plane of graphite, respectively. The element mappings (Figure 3d and Figure 3e) show that C and N were evenly distributed in the selected area. In other words, N-doped carbon was formed as a support material. The images also show that Fe and Ni uniformly covered the entire particles, forming Fe and Ni alloy particles.

**Figure 4:**
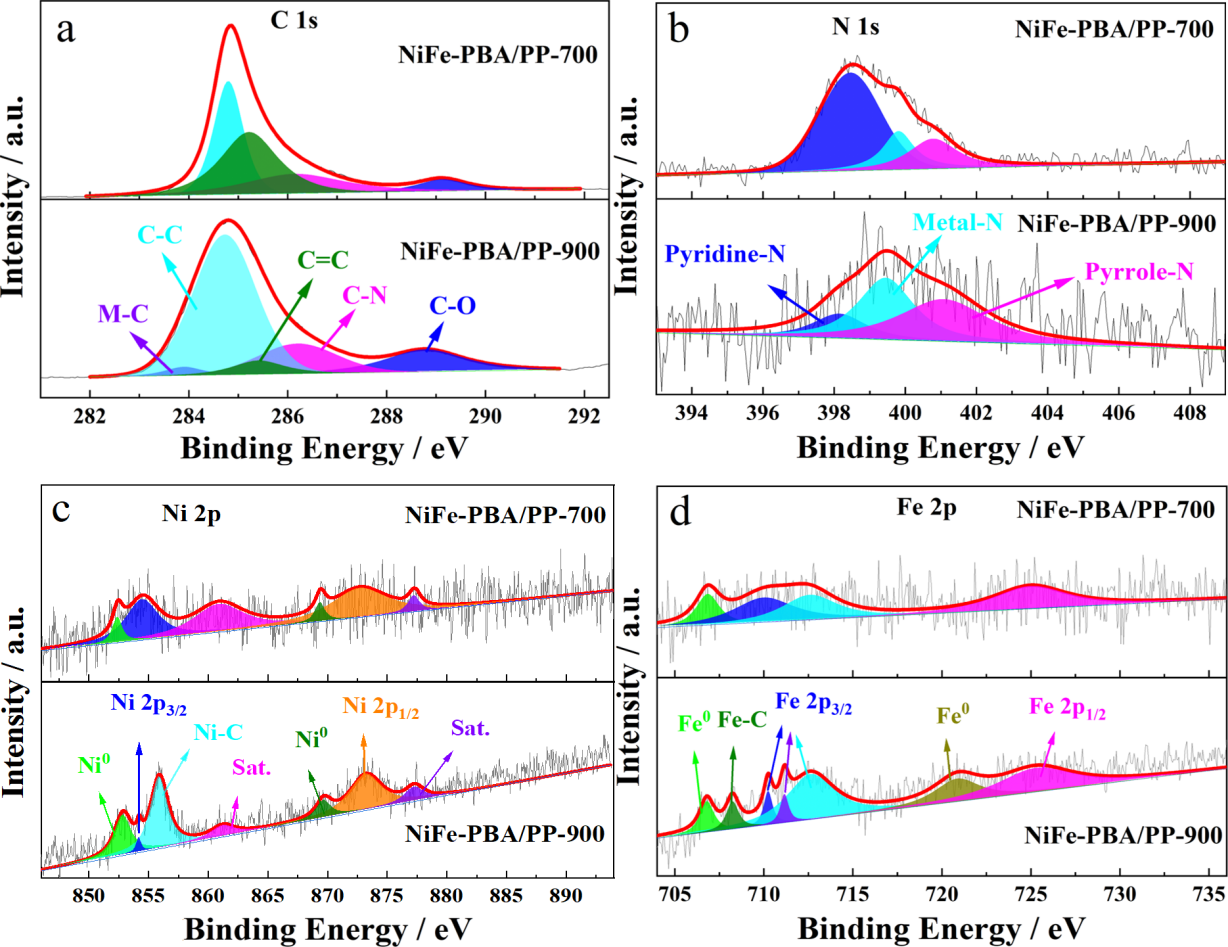
High-resolution XPS spectra of NiFe-PBA/PP-T calcined at 700 (top panels) and 900 °C (bottom panels) for (a) C 1s, (b) N 1s, (c) Ni 2p, and (d) Fe 2p.

To gain further information on the chemical state and element composition on the surface of NiFe-PBA/PP-T samples, NiFe-PBA/PP-T was investigated via XPS (Figure 4). In the C 1s XPS spectra of NiFe-PBA/PP-T (Figure 4a), the binding energy values of 284.8, 285.3, 286.2, and 289 eV correspond to C–C, C=C, C–N, and C–O species, respectively [29,47–48]. For NiFe-PBA/PP-900, the peak at 283.2 eV corresponds to metal–carbon bonds, which indicates the formation of transition metal carbides at a higher calcination temperature. As shown in Figure 4b, the N 1s XPS spectrum consists of three main peaks at 398.5, 399.8 and 400.8 eV, corresponding to pyridinic nitrogen, metal–nitrogen, and pyrrolic nitrogen [49], respectively. The fitted Ni 2p XPS spectrum of NiFe-PBA/PP-900 (Figure 4c) shows peaks at 852.9 and 869.7 eV, which correspond to Ni 2p_3/2_ and Ni 2p_1/2_ of Ni^0^, respectively, and two peaks at 854.5 and 873.2 eV, which correspond to Ni 2p_3/2_ and Ni 2p_1/2_ of Ni^2+^ (corresponding to NiC), respectively. This confirms that nickel carbide was formed on these samples. However, two peaks that correspond to nickel carbide were not observed in the Ni 2p XPS spectrum of NiFe-PBA/PP-700 [29]. Compared to the Fe 2p XPS spectrum of NiFe-PBA/PP-700 (Figure 4d), and apart from the peaks related to Fe 2p_1/2_ and Fe 2p_3/2_ of Fe^2+^ and Fe^3+^, respectively, a new peak at 708.2 eV was observed in the Fe 2p XPS spectrum of NiFe-PBA/PP-900. This new peak at 708.2 eV was indexed to the Fe 2p_3/2_ of iron carbide [50]. Figure 4 indicates that nickel carbide was formed on NiFe-PBA/PP when it was heated to 900 °C.

**Figure 5:**
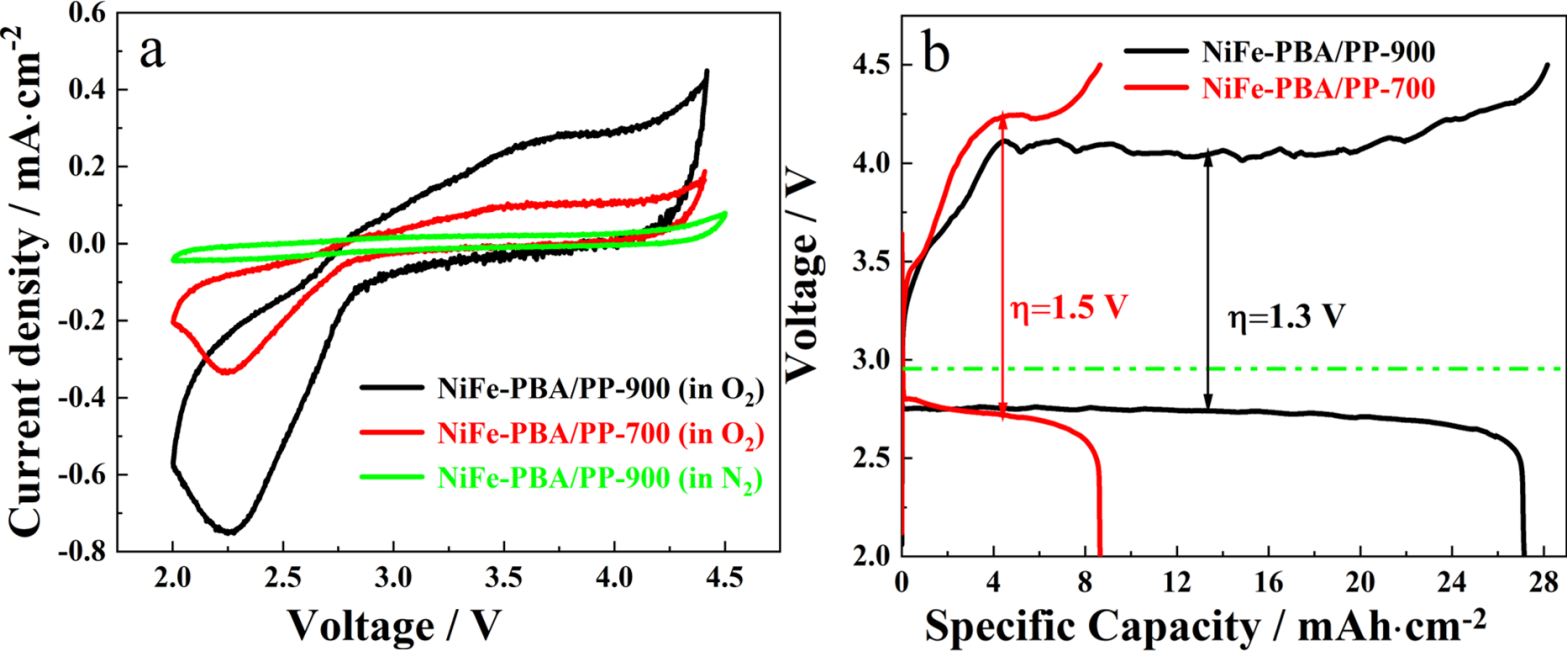
(a) In situ cyclic voltammetry curves of Li–O_2_ batteries with various cathodes. (b) Charge and discharge curves of Li–O_2_ batteries with NiFe-PBA/PP-T calcined at 700 and 900 °C.

The electrocatalytic properties of NiFe-PBA/PP-T samples were initially evaluated by cyclic voltammetry. As shown in Figure 5a, the current response in N_2_ was very low, close to zero, during positive and negative scans. This suggests that the as-prepared cathode has no ability to catalyze N_2_ reduction. In contrast, both samples exhibit an oxygen reduction peak on the positive scan in O_2_, indicating that these materials have good catalytic activity towards ORR. Figure 5a shows that the ORR onset cell voltage of NiFe-PBA/PP-900 (2.86 V) is higher than that of NiFe-PBA/PP-700 (2.78 V), and the current density of the cathodic peak for NiFe-PBA/PP-900 is also much higher than that of NiFe-PBA/PP-700. The CV result confirms that NiFe-PBA/PP-900 has a superior catalytic activity towards ORR than NiFe-PBA/PP-700. In addition, the current density of the OER for NiFe-PBA/PP-900 is significantly higher than that for NiFe-PBA/PP-700. The integrated area under the CV curve of NiFe-PBA/PP-900 is also much larger than that of NiFe-PBA/PP-700, suggesting that NiFe-PBA/PP-900 has a higher specific capacity than NiFe-PBA/PP-700. Galvanostatic charge and discharge experiments were carried out to investigate the electrocatalytic performance of NiFe-PBA/PP-T samples as the cathode material in Li–O_2_ cells (Figure 5b). All obtained current values were normalized to the area of the cathode. The initial charge and discharge experiments were performed at a current density of 0.1 mA·cm^−2^. As shown in Figure 5b, the specific discharge capacity values of NiFe-PBA/PP-900 and NiFe-PBA/PP-700 were 27.14 and 8.64 mA·cm^−2^, respectively. Both materials show a stable discharge voltage plateau, specifically NiFe-PBA/PP-900, which delivered a discharge voltage plateau of 2.75 V with an overpotential of 0.21 V. Under our conditions, it was found that the overpotential of NiFe-PBA/PP-900 in the Li–O_2_ cell was comparable to data reported in the literature, as shown in Table 1. An ideal Li–O_2_ cell has a low charge voltage plateau and a high discharge voltage plateau. The gap between charge and discharge voltage plateaus of the Li–O_2_ battery with a NiFe-PBA/PP-900 cathode was 1.3 V, which is lower than that of NiFe-PBA/PP-700 (1.5 V). In other words, NiFe-PBA/PP-900 has a better cell performance than NiFe-PBA/PP-700. This improved electrocatalytic activity was probably due to the formation of nickel carbide at 900 °C with an expanded d-band structure of nickel, which arised from the synergistic effect between nickel and carbon in NiC*_x_*.

**Table 1:** Performance comparison of Li–O_2_ batteries.

cathodes	current density	specific capacity	cycle stability	references

CoNi/N-graphene	0.5 mA·cm^−2^	2.156 mAh·cm^−2^	100 (limited capacity: 0.24 mAh·cm^−2^)	[14]
Ni/GF	100 mA·g^−1^	22035 mAh·g^−1^	34	[51]
Mo_2_C	0.08 mA·cm^−2^	2.87 mAh·cm^−2^	124 (limited capacity: 0.25 mAh·cm^−2^)	[52]
Mo_2_C/N-C	0.04 mA·cm^−2^	3.08 mAh·cm^−2^	200 (limited capacity: 0.25 mAh·cm^−2^)	[53]
Ru/wood-C	0.1 mA·cm^−2^	8.58 mAh·cm^−2^	100 (limited capacity: 0.6 mAh·cm^−2^)	[54]
3DP-NC-Co	0.05 mA·cm^−2^	14.6 mAh·cm^−2^	—	[55]
CNT@Ni@NiCo silicate	0.03 mA·cm^−2^	1.51 mAh·cm^−2^	60 (limited capacity: approx. 0.21 mAh·cm^−2^)	[56]
NiFe@NC/PPC	0.1 mA·cm^−2^	13.79 mAh·cm^−2^	290 (limited capacity: 0.3 mAh·cm^−2^)	[40]
this work	0.1 mA·cm^−2^	27.14 mAh·cm^−2^	145 (limited capacity: 0.5 mAh·cm^−2^)	—

**Figure 6:**
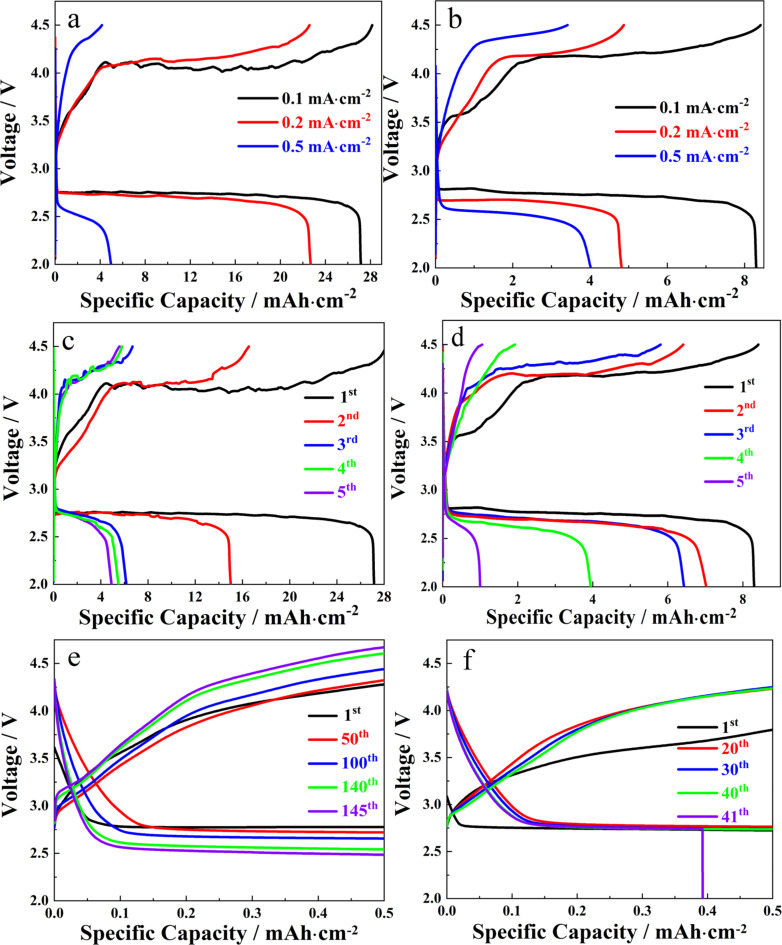
Rate performance of the (a) NiFe-PBA/PP-900 cathode and of the (b) NiFe-PBA/PP-700 cathode at 0.1, 0.2, and 0.5 mA·cm^−2^. The battery performance of the (c) NiFe-PBA/PP-900 cathode and of the (d) NiFe-PBA/PP-700 cathode under full charge and discharge cycles at 0.1 mA·cm^−2^ (1st to 5th). The cycling stability of (e) the NiFe-PBA/PP-900 cathode (1st to 145th) and of (f) the NiFe-PBA/PP-700 cathode (1st to 41th) at 0.1 mA·cm^−2^ with a limited capacity of 0.5 mAh·cm^−2^.

Figure 6a shows the rate performance of NiFe-PBA/PP-900 at current density values ranging from 0.1 to 0.5 mA·cm^−2^ within a cell voltage window ranging from 2.0 to 4.5 V. It was observed that, when the current density increased five-fold, the specific discharge capacity of the NiFe-PBA/PP-900 cell went from 27.14 to 4.95 mAh·cm^−2^. The cycling stability of NiFe-PBA/PP-900 in the Li–O_2_ cell was analyzed by performing full charge and discharge cycles at 0.1 mA·cm^−2^ varying the voltage window from 2.0 V to 4.5 V. By comparison, NiFe-PBA/PP-700 only delivered a specific capacity of 8.2 mAh·cm^−2^ at 0.1 mA·cm^−2^ and decreased sharply to 4.0 mAh·cm^−2^ when the current density increased to 0.5 mA·cm^−2^ (Figure 6b). Figure 6c and Figure 6d show the cycling stability of Li–O_2_ batteries with NiFe-PBA/PP-T cathodes under full charge and discharge cycles at 0.1 mA·cm^−2^. The specific capacity of NiFe-PBA/PP-700 and NiFe-PBA/PP-900 decreased gradually. However, the overpotential increased with the increase in the number of cycles. This can be attributed to the undecomposed Li_2_O_2_ covering the surface of the electrode. Moreover, the decomposition of the electrolyte at a high voltage is another possible reason, resulting in the decrease of the capacity during the cycling test. However, the specific capacity of NiFe-PBA/PP-900 remained at 4.9 mAh·cm^−2^ after the cell was cycled for five times (Figure 6c). This value is almost four times higher than that of NiFe-PBA/PP-700 (Figure 6d).

The cycling stability of NiFe-PBA/PP-T was further evaluated at a current density of 0.1 mA·cm^−2^ and with a limited capacity of 0.5 mAh·cm^−2^ (Figure 6e and Figure 6f). With the charge and discharge cycles carried out under the limited capacity value, NiFe-PBA/PP-900 demonstrated a good cycling stability. As shown in Figure 6e, the discharge cell voltage plateau was very stable from the first to the 145th cycle. The charge cell voltage increased with the cycling number, probably due to the incomplete decomposition of Li_2_O_2_. After the 100th cycling test, the discharge cell voltage plateau decreased from 2.78 to 2.5 V. It can be observed that the NiFe-PBA/PP-900 cell exhibited a cycling stability superior to that of the NiFe-PBA/PP-700 cell. The NiFe-PBA/PP-700 cell stopped working at the 41st cycle (Figure 6f). Table 1 compares the performance of the cathodes synthesized here with other cathodes for Li–O_2_ batteries. A superior performance highlights the good catalytic activity of NiFe-PBA/PP-900. Furthermore, when compared with a similar cathode reported in our previous article, the specific capacity of NiFe-PBA/PP-900 is almost twice as high as that of NiFe@NC/PPC at the same current density [40]. This is probably due to the synergistic effects between NiFe alloy, transition carbides, and N-doped carbon materials. Akhtar et al. [29] suggested that the presence of Ni and electron-rich nanointerfaces across the Ni–Ni_3_C interface seems to facilitate the process of adsorption/desorption of intermediate species and the charge transfer ability during hydrogen evolution reaction (HER)/OER. In addition, the interfaces between Ni_3_C and N-doped C probably strongly reshuffle the electronic density, resulting in enhanced catalytic activity. In this work, during hydrothermal pretreatment, the ion-exchange reaction of OH^−^/[Fe(CN)_6_]^3−^ occurred at the interface between NiFe-PBA cubes and NaOH solution, resulting in Ni(OH)_2_/NiFe-PBA core–shell structure [44–46]. During the calcination process, Ni(OH)_2_ was converted to NiC*_x_*, and the NiFe-PBA core was converted to the NiFe alloy coated by N-doped carbon. Compared with the composite structure of the NiFe alloy and N-doped carbon in NiFe@NC/PPC [40], the introduction of NiC*_x_* may bring more heterointerfaces [29], which may be the source of enhanced performance.

**Figure 7:**
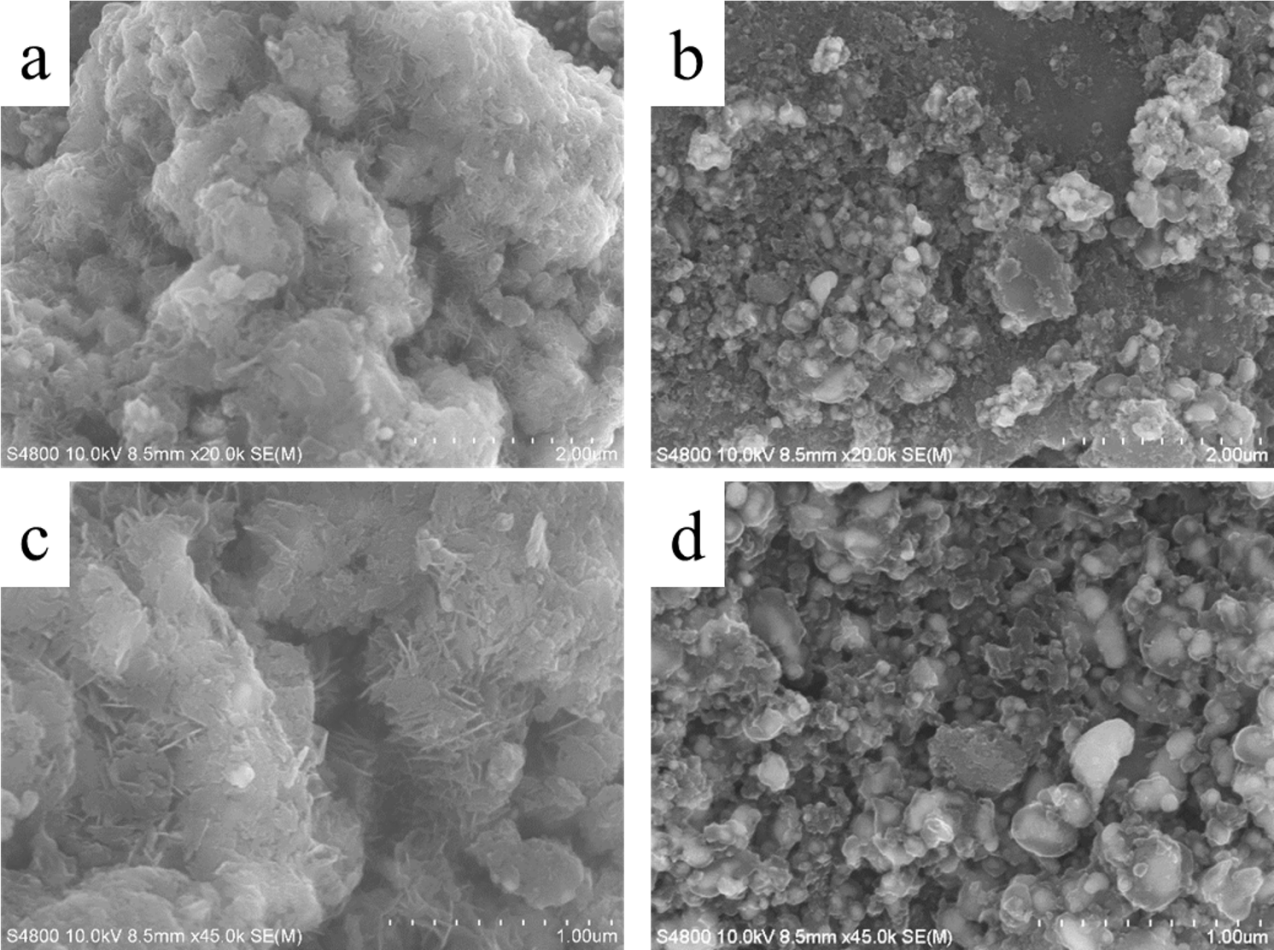
(a,c) SEM images of NiFe-PBA/PP-900 cathodes after the first discharge and (b,d) after the first charge.

The reversibility and the decomposition of the product during discharge were measured with SEM (Figure 7). An SEM image of the NiFe-PBA/PP-900 cathode after the first discharge was taken after the Li–O_2_ cell was discharged to 2.0 V at 0.1 mA·cm^−2^. Compared to the grape-like morphology of fresh NiFe-PBA/PP-900 (Figure 2c), larger lumps were observed in the SEM image of NiFe-PBA/PP-900 after the first discharge (Figure 7a and Figure 7c). The surfaces of the grape-like particles were completely covered by newly formed Li_2_O_2_. It was reported that Li_2_O_2_ with a film-like morphology is easily decomposed at a low charge voltage in comparison to its crystalline counterpart, since the film-like Li_2_O_2_ has an improved ionic conductivity [57]. After the first charge, the obtained SEM images (Figure 7b and Figure 7d) show that most of the newly formed Li_2_O_2_ was decomposed. However, a small amount of Li_2_O_2_ still remained on the surface of the catalyst particles. These undecomposed Li_2_O_2_ might be attributed to the formation of crystalline Li_2_O_2_, which is probably the main reason for the observed decline in the specific capacity (Figure 6c).

**Figure 8:**
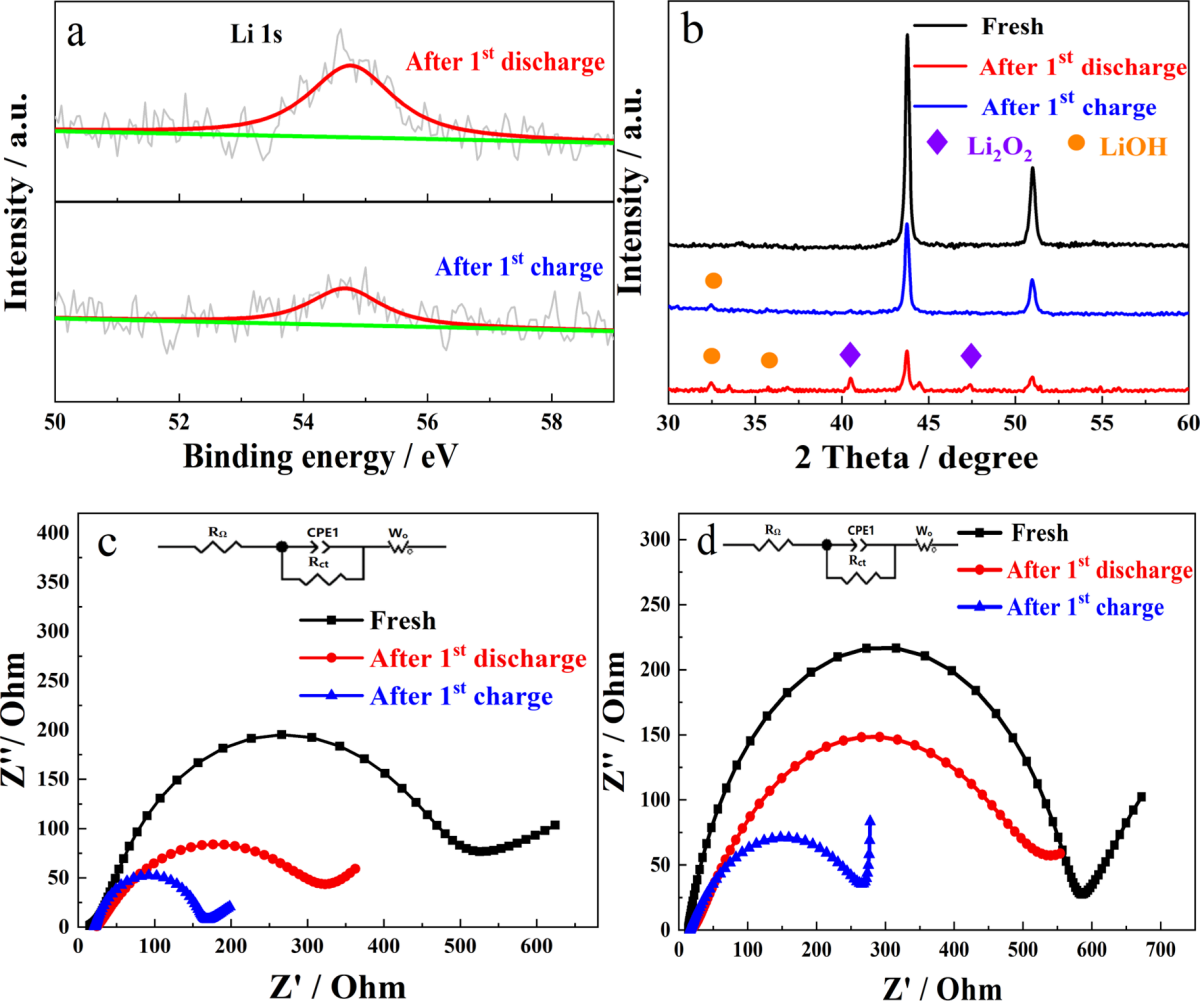
(a) High-resolution Li 1s XPS spectra of NiFe-PBA/PP-900 after the 1st full discharge and the 1st full charge at 0.1 mA·cm^−2^. (b) XRD patterns of fresh NiFe-PBA/PP-900 after the 1st full discharge and the 1st full charge at 0.1 mA·cm^−2^. Electrochemical impedance spectra of (c) NiFe-PBA/PP-900 and of (d) NiFe-PBA/PP-700.

To identify the product formed on the surface of NiFe-PBA/PP-900 after charge and discharge, Li 1s XPS was used to analyze these samples. Figure 8a shows a sharp Li 1s peak at approx. 54.7 eV after the first discharge, which suggests that the main product formed during the discharge was Li_2_O_2_ [58]. The intensity of the Li 1s peak decreased after the first discharge. However, the peak did not completely disappear, indicating that there was still some Li_2_O_2_ remaining on the surface of NiFe-PBA/PP-900. In addition, XRD was also performed to identify the product formed on the surface of NiFe-PBA/PP-900 after charge and discharge, as shown in Figure 8b. It was confirmed that the main discharge product was Li_2_O_2_ (JCPDF 09-0355). It is noteworthy that LiOH was also detected. This can be associated with the reaction between Li_2_O_2_ and H_2_O when the discharged/recharged electrode was exposed to air during XRD testing. After charging to 4.5 V, trace amounts of LiOH were detected, due to the existence of some amount of undecomposed Li_2_O_2_. Electrochemical impedance spectroscopy was also carried out to investigate NiFe-PBA/PP-T samples after the first charge and discharge (Figure 8c and Figure 8d). The illustrations (top part of Figure 8c and Figure 8d) show the corresponding equivalent circuit. The ohmic resistance (*R*_Ω_) and charge-transfer resistance (*R*_ct_) values of the fresh sample and of the sample after the first charge and first discharge were obtained by analyzing the EIS curves (Table 2). The value of *R*_Ω_ of NiFe-PBA/PP-T after the first discharge was higher than that of the fresh sample due to the relatively low electronic conductivity of Li_2_O_2_ formed on the surface of the cathode. After the first charge, *R*_Ω_ was still higher than that of the fresh sample, but lower than that of the sample after the first discharge, indicating that there was still some remaining Li_2_O_2_. As shown in Figure 8c and Figure 8d, *R*_ct_ of NiFe-PBA/PP-T after the first discharge was lower than that of the fresh sample, possibly due to an improvement in the contact between NiFe-PBA/PP-T and the electrolyte formed during the discharge process. Since the contact between the active material and the electrolyte was further improved during the electrochemical process, *R*_ct_ of NiFe-PBA/PP-T after the first charge was lower than that of NiFe-PBA/PP-T after the first discharge. Furthermore, *R*_Ω_ and *R*_ct_ (Table 2) of NiFe-PBA/PP-900, at different stages, are lower than that of NiFe-PBA/PP-700, which reveals an enhanced catalytic activity of NiFe-PBA/PP-900.

**Table 2:** Fitted impedance parameters of Li–O_2_ batteries with NiFe-PBA/PP-900 and NiFe-PBA/PP-700 at different stages.

NiFe-PBA/PP-700	*R*_Ω_ (Ω)	*R*_ct_ (Ω)	NiFe-PBA/PP-900	*R*_Ω_ (Ω)	*R*_ct_ (Ω)

fresh	23.5	559.1	fresh	14.4	470.5
after 1st discharge	26.4	437.6	after 1st discharge	22.6	259.3
after 1st charge	25.1	267.6	after 1st charge	21.2	138.5

The above findings indicate that the binder-free NiFe-PBA/PP-T cathode exhibited superior ORR electrocatalytic performance in Li–O_2_ cells. Compared to NiFe-PBA/PP-700, the sample heated to 900 °C showed an improved electrocatalytic activity and durability towards ORR. The enhanced performance of NiFe-PBA/PP-900 was due to several reasons. The first reason is the hybridization between the d-orbital of Ni and s- and p-orbitals of carbon in NiC*_x_* formed at 900 °C, which yielded NiFeC containing NiFe alloy and NiC*_x_* and exhibited a superior electrocatalytic performance as a result from the synergistic effect between these components. The second reason is that grape-like nanoparticles of NiFeC supported on porous N-doped carbon derived from PP provided more exposed active sites for ORR. The third reason is that there were NiFe alloy and NiC*_x_* materials inside the NiFeC nanoparticles formed on the surface of N-doped carbon. These structures efficiently improved the electron and ion transfer between the cathode and electrolyte during charging and discharging processes. Finally, NiFeC nanoparticles supported on N-doped carbon significantly impeded particle aggregation during the electrochemical reactions, leading to a relatively high stability. The NiFe-PBA/PP-900 developed in this study suggests that inexpensive and economically viable ORR catalysts for Li–O_2_ cells are achieved by rationally designing the morphology and tuning the composition of the catalysts.

## Conclusion

In this investigation, heterostructured NiC*_x_*-NiFe-NC derived from bimetallic Prussian blue supported on biochar was successfully synthesized to improve the electronic conductivity and electrocatalytic activity, and was used as the cathode material for Li–O_2_ batteries. When the precursor was heated to 900 °C, the metal carbides were formed on the N-doped carbon, which played a critical role in improving the electrocatalytic performance of Li–O_2_ batteries. NiFe-PBA/PP-900 delivered a specific discharge capacity of 27.14 mAh·cm^−2^ at 0.1 mA·cm^−2^. The enhanced electrocatalytic performance was due to the synergistic effects between the NiFe alloy and metal carbides inside the nanoparticles derived from Prussian blue. This study indicates that carbides formed on porous N-doped carbon may lead to a promising strategy to achieve high capacity and cyclability of Li–O_2_ batteries.
